# The Impact of Warming and Nutrients on Algae Production and Microcystins in Seston from the Iconic Lake Lesser Prespa, Greece

**DOI:** 10.3390/toxins10040144

**Published:** 2018-04-02

**Authors:** Valentini Maliaka, Elisabeth J. Faassen, Alfons J.P. Smolders, Miquel Lürling

**Affiliations:** 1 Institute for Water and Wetland Research, Department of Aquatic Ecology and Environmental Biology, Radboud University, Heyendaalseweg 135, 6525 AJ Nijmegen, The Netherlands; a.smolders@science.ru.nl; 2 Aquatic Ecology & Water Quality Management Group, Department of Environmental Sciences, Wageningen University, PO Box 47, 6700 AA Wageningen, The Netherlands; els.faassen@wur.nl (E.J.F.); miquel.lurling@wur.nl (M.L.); 3 Society for the Protection of Prespa, Agios Germanos 53077, Greece; 4 Research Institute RIKILT, BU Contaminants & Toxins, Wageningen University, Akkermaalsbos 2, 6708 WB Wageningen, The Netherlands; 5 B-WARE Research Centre, Radboud University, Toernooiveld 1, 6525 ED Nijmegen, The Netherlands; 6 Department of Aquatic Ecology, Netherlands Institute of Ecology (NIOO-KNAW), P.O. Box 50, 6700 AB Wageningen, The Netherlands

**Keywords:** bioassay, climate change, cyanotoxins, eutrophication, nutrient addition.

## Abstract

Lake Lesser Prespa and its adjacent pond, Vromolimni in Greece, is a shallow freshwater system and a highly protected area hosting an exceptional biodiversity. The occurrence of microcystins (MCs) producing cyanobacterial blooms in these waters during recent years can be harmful to the wildlife. We tested the hypothesis that both cyanobacterial biomass and MCs are strongly influenced by nutrients (eutrophication) and warming (climate change). Lake and pond water was collected from two sites in each water body in 2013 and incubated at three temperatures (20 °C, 25 °C, 30 °C) with or without additional nutrients (nitrogen +N, phosphorus +P and both +N and +P). Based on both biovolume and chlorophyll-a concentrations, cyanobacteria in water from Lesser Prespa were promoted primarily by combined N and P additions and to a lesser extent by N alone. Warming seemed to yield more cyanobacteria biomass in these treatments. In water from Vromolimni, both N alone and N+P additions increased cyanobacteria and a warming effect was hardly discernible. MC concentrations were strongly increased by N and N+P additions in water from all four sites, which also promoted the more toxic variant MC-LR. Hence, both water bodies seem particularly vulnerable to further N-loading enhancing MC related risks.

## 1. Introduction

Lake Lesser Prespa (47.4 km^2^) and its adjacent ponds are part of the Prespa Lake basin and located in the North-western part of Greece (South-Eastern Europe). The Prespa Lakes area is recognized as a wetland of great international importance (Ramsar site) and is a Special Protected Area [Directive 79/409/EEC] [1]. Its high endemic biodiversity and being a refuge for many migratory birds, among them the iconic piscivorous Great White Pelican (*Pelecanus onocrotalus*) and the Dalmatian Pelican (*Pelecanus crispus*)—which has one of the largest colonies worldwide in the lake [1,2]—give the area a high ecological value. The traditional way of fishing is one of the socio-cultural values of the area, while the consumption of fish, the use of water for irrigation and ecotourism are direct economic values provided by the lake (cf. [3]).

The water quality of Lake Lesser Prespa is, however, under anthropogenic pressure through discharge of communal waste water, irrigation and run-off from intensive agricultural practices and sedimentation of eroded matter [1]. Besides, water-bird-derived nutrients (guanotrophication) supplied by the most abundant breeding species (pelicans, cormorants) are expected to contribute further to the eutrophication of the lake, particularly on a local-scale, near their colonies [4]. Guanotrophication is already reflected by the excessive chlorophyll-a and nutrient levels which have been measured within the small isolated Vromolimni Pond (0.25 km^2^), where large mixed populations of pelicans and cormorants breed annually [1,5]. In the Prespa basin altered rainfall patterns and ongoing water abstractions are also expected to speed-up eutrophication [6]. The resulting over-enrichment of the water with nutrients is leading to cyanobacterial blooms and in a recent study, the occurrence of occasionally high microcystin concentrations (MC) in the lake—as well as MC in the tissue of carp, pelicans and otters—was determined [5]. Such cultural eutrophication is one of the main drivers causing excessive phytoplankton growth and nuisance algal blooms worldwide [7,8,9] but also global warming is predicted to stimulate proliferation of cyanobacterial blooms [9,10,11]. Warming and warming-enhanced nutrient loading are projected to act in synergy in intensifying eutrophication symptoms [12]. Furthermore, model forecasts suggest that warming-augmented run-off and influx of nutrients will increase total biomass of the cyanobacterial communities [13]. 

Cyanobacterial communities consist of toxic and non-toxic species and within the toxic species often toxigenic and non-toxigenic strains co-occur [14]. Warming and eutrophication seem to benefit the toxigenic strains more than non-toxigenic strains [15], however, there is no clear relation between the proportion of toxigenic genotypes and actual surface water cyanotoxin (MC) concentrations [14,16]. This is due to the fact that surface water MC concentrations are not only determined by biomass of toxigenic cells but also by the toxin quota of those cells, where these quotas depend on environmental factors affecting MC synthesis and fate [16]. In a recent experiment a pulse of nutrients and warming increased cyanobacterial biomass and MC concentrations in water collected from a eutrophic pond but MC quota per unit biomass seemed to decrease [17]. Based on that experiment, we hypothesize that cyanobacteria and MCs will be promoted under warmer conditions and increased availability of nutrients in the seston from Lake Lesser Prespa and the Vromolimni Pond. Furthermore, we assumed that the limiting nutrient (nitrogen versus phosphorus) might differ between the very eutrophic Vromolimni Pond and the rather mesotrophic Lake Lesser Prespa. To this end, we tested the effects of warming at three different temperatures (20 °C, 25 °C, 30 °C) both without adding extra nutrients and with adding nitrogen (N), phosphorus (P) or both N and P. 

## 2. Results 

### 2.1. Initial Chlorophyll-a, Nutrient and MC Concentrations in Water from Lake Lesser Prespa and Vromolimni Pond

**Figure 1:**
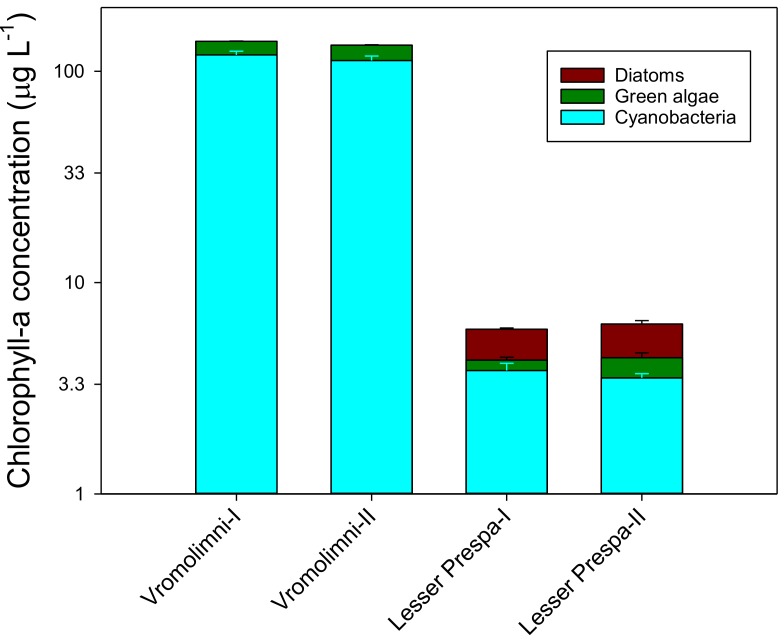
Initial Chlorophyll-a measurements at the onset of the algal bioassay (*t* = 0) at water samples collected from Vromolimni Pond and Lesser Prespa Lake in late September 2013.

The initial chlorophyll-a measurements, determined with PHYTO-PAM at the onset of the incubation period (*t* = 0) show that cyanobacterial chlorophyll had the major contribution to the total chlorophyll-a in the surface water samples from Vromolimni Pond and Lake Lesser Prespa (up to 86.2% and 63.7% respectively) (Figure 1). Cyanobacterial chlorophyll and total chlorophyll-a concentrations were much higher in Vromolimni Pond than in samples from Lake Lesser Prespa. Post-hoc comparison (the Holm-Sidak Method) revealed two homogeneous groups: 1) the two sites from Vromolimni and 2) the sites from Lesser Prespa (Figure 1). Chlorophyll-a in water from Vromolimni was 85% cyanobacterial and 15% from eukaryotic algae (green algae), while this was 55% and 45%, respectively in water from Lesser Prespa (Figure 1). No diatom chlorophyll was detected in the samples from Vromolimni Pond. Field measurements (determined with an AlgaeTorch10) in late September 2013 also showed that most of the total chlorophyll-a in Vromolimni Pond (ranging between 126.9–186.3 µg L^−1^) was cyanobacterial (91.8–130 µg L^−1^). Much lower chlorophyll-a concentrations were measured in Lake Lesser Prespa (7.4–27.3 µg L^−1^) at that time, where cyanobacterial chlorophyll ranged between 5.3–17.3 µg L^−1^ at water temperatures ranging from 18.2–21.2 °C. *Microcystis* sp. were commonly found in the water samples from Vromolimni Pond and Lake Lesser Prespa and *Aphanocapsa* sp. and *Dolichospermum* sp. were also found but in lower abundances. 

The total MC concentrations in Vromolimni I and II were 2.4 and 1.9 µg L^−1^, respectively, while in Lesser Prespa I and II it was 0.8 µg L^−1^. The samples were also analysed for nodularin but no measurable levels were detected. Dissolved phosphate concentrations in Vromolimni I and II were 79 and 45 µg P L^−1^, respectively, while in Lesser Prespa I and II they were 35 and 4 µg P L^−1^. Dissolved Inorganic Nitrogen (DIN) concentrations were 18 µg N L^−1^ in Lesser Prespa I and II, 34 µg P L^−1^ in Vromolimni I and 39 µg P L^−1^ in Vromolimni II. DIN:P ratios were 0.5 and 4.5 in Lake Lesser Prespa I and II and 0.4 and 0.9. in Vromolimni Pond I and II As a proxy for the phytoplankton limiting nutrient, these inorganic N:P ratios indicate N-limiting conditions [18].

### 2.2. Final Chlorophyll-a and Biovolume Concentrations 

**Figure 2:**
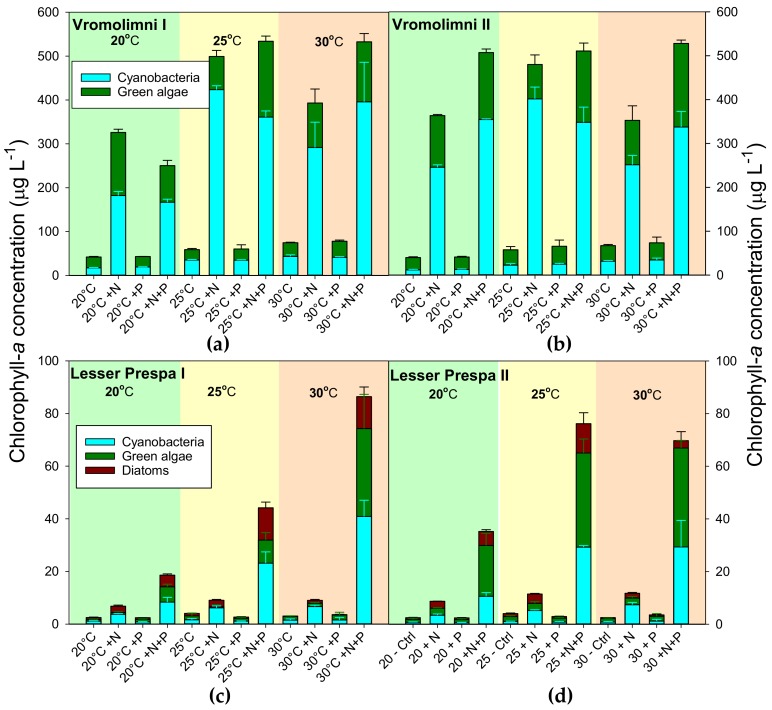
PHYTO-PAM measurements of cyanobacterial (blue), green algal (green) and diatoms (brown) chlorophyll-a concentrations (µg L^−1^) in surface water taken from two locations in Vromolimni Pond (Panels **a** and **b**) and two in Lake Lesser Prespa (Panels **c** and **d**) after seven days incubation at regular (20 °C), high (25 °C) and extreme (30 °C) temperatures without extra nutrients added (Control, Ctrl), solely N addition (+N), solely P addition (+P) and both N+P addition (+N+P). Error bars indicate one standard deviation (*n* = 3).

Incubating water from Vromolimni clearly yielded higher chlorophyll-a concentrations in the +N and +N+P treatments (Figure 2a,b). In water from site I this increase seemed somewhat lower at 20 °C than at 25 °C and 30 °C (Figure 2a), however, in water from site II such a pattern was not that clear (Figure 2b). Three-way and separate two-way ANOVAs could not be performed due to non-normal distribution of data that could not be overcome by transformations. Hence, non-parametric Kruskal-Wallis One Way Analysis of Variance on Ranks were run instead, which indicated significant differences in incubations from the Vromolimni site I (*p* < 0.001) and site II (*p* < 0.001). Tukey’s post-hoc comparison revealed that for both locations, the 20 °C incubations were different from the 25 °C +N+P and 30 °C +N+P treatments and also that in the series with water from Vromolimni site I the 20 °C +P treatments were different from the 25 °C +N+P treatments. 

Incubations with water from the two sites in Lesser Prespa visibly yielded higher chlorophyll-a concentrations in the +N+P treatments than in the other treatments (Figure 2c,d). In water from Lesser Prespa I these +N+P treatments expressed an interaction with temperature (Figure 2c), while in water from site II only the chlorophyll-a concentrations in the 20 °C +N+P treatments seemed lower than those in the 25 °C +N+P and 30 °C +N+P treatments (Figure 2d). Chlorophyll-a concentrations in +N treatments also seemed somewhat elevated compared to non-treated or solely +P-treated water (Figure 2c,d). Kruskal-Wallis One Way Analysis of Variance on Ranks indicated significant differences in incubations from Lesser Prespa site I (*p* < 0.001), where Tukey’s post-hoc comparison revealed that 20 °C and 25°C +P treatments were different from the 30 °C +N+P treatments (Figure 2c). Likewise, differences among incubations with water from site II were detected (*p* < 0.001) and post-hoc tests revealed that only the 20 °C +P and 25 °C +N+P treatment differed from each other (Figure 2d).

Cyanobacterial chlorophyll concentrations followed the same pattern as total chlorophyll-a with clearly higher concentrations in the +N and +N+P treatments (Figure 2). Total- and cyanobacterial chlorophyll-a concentrations were higher in the Vromolimni incubations than in the incubations with water from Lesser Prespa (Figure 2). 

In all the non-treated and +P treated incubations cyanobacterial growth rates (based on chlorophyll-a) were negative meaning a decline in cyanobacterial chlorophyll concentrations over the incubation period (Appendix A). In the Vromolimni incubations all eukaryotic algal growth rates were positive. In all incubations, eukaryotic algal growth rates were higher or less negative than the cyanobacterial growth rates (Appendix A).

**Figure 3:**
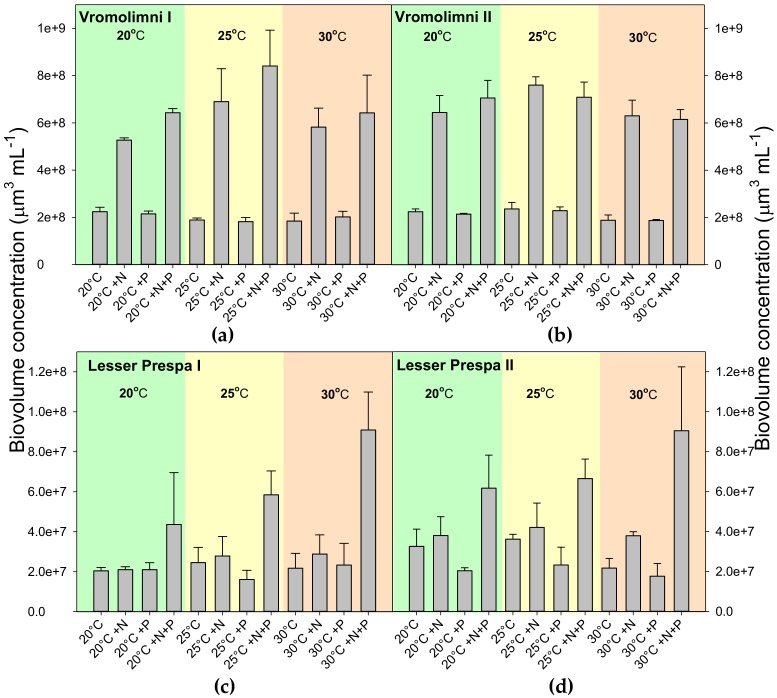
Biovolume concentrations (µm^3^ mL^−1^) in surface water taken from two locations in Vromolimni Pond (Panels **a** and **b**) and two in Lake Lesser Prespa (Panels **c** and **d**) after seven days incubation at regular (20 °C), high (25 °C) and extreme (30 °C) temperatures without extra nutrients added (Control, Ctrl), solely N-addition (+N), solely P addition (+P) and both N+P addition (+N+P). Error bars indicate one standard deviation (*n* = 3).

Similar to chlorophyll-a concentrations, overall, biovolume concentrations after seven days of incubation, were much higher in water from Vromolimni than in water from Lesser Prespa (Figure 3).For the Vromolimni sites biovolume concentrations were higher in + N and +N+P treatments while water from Lesser Prespa responded most strongly to +N+P treatments (Figure 3). 

### 2.3. Final Microcystin Concentrations

**Figure 4:**
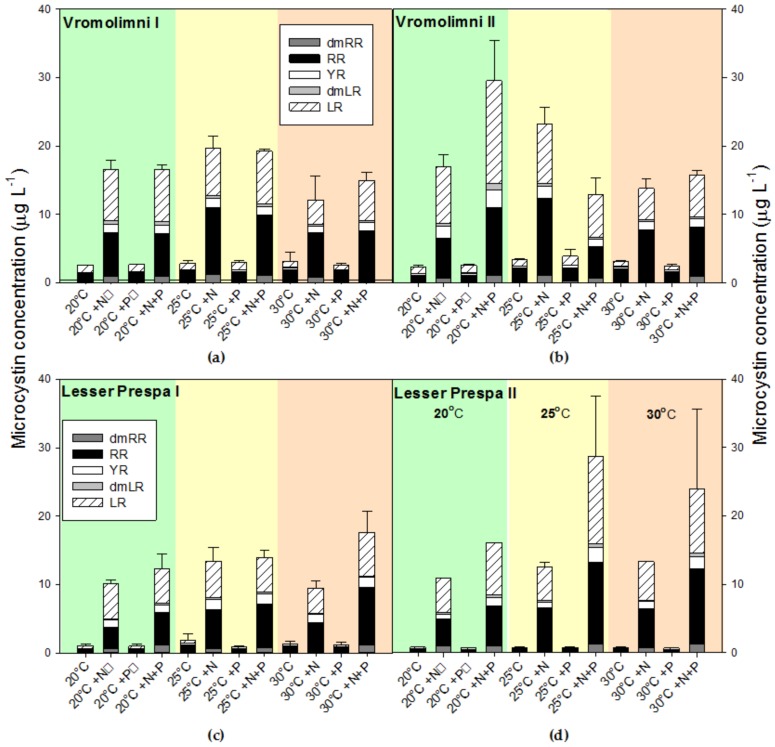
Concentrations of five different microcystin variants (µg L^−1^) in surface water taken from two locations in Vromolimni Pond (Panels **a** and **b**) and two in Lake Lesser Prespa (Panels **c** and **d**) after seven days incubation at regular (20 °C), high (25 °C) and extreme (30 °C) temperatures without additional nutrients added, solely N-addition (+N), solely P-addition (+P) and both N+P addition (+N+P). Error bars indicate one standard deviation (*n* = 3).

Microcystins (MCs) were detected in all analysed samples and, more specifically, five different variants were found: dmRR, RR, YR, dmLR and LR (Figure 4). The MC variant RR was present in all samples, YR and LR in 98.5% of the samples, dmLR in 94% of the samples and dmRR in 35% of the samples. In all incubations with +N addition or +N+P addition the total MC concentrations were higher than in the non-treated or solely +P-treated incubations (Figure 4). For incubations with water from Vromolimni I log transformation did not result in meeting ANOVA requirements (Shapiro-Wilk normality test failed, *p* < 0.05) and no statistical test was performed. In water from Vromolimni II, however, a two-way ANOVA indicated a significant treatment effect (*p* < 0.001), a significant temperature effect (*p* = 0.007) and a significant treatment x temperature interaction (*p* < 0.001). The latter means that the effect of treatments differed per incubation temperature, which is for instance visible in total MC concentrations being higher in the +N+P treatment than the +N treatment at 20 °C, while this was the opposite at 25 °C (Figure 4b). Tukey’s test revealed four homogenous groups: 1) all non-treated and P-treated incubations, 2) 20 °C +N+P, 3) 25 °C +N and 4) 20 °C +N, 25 °C +N+P, 30 °C +N and 30 °C +N+P.

A two-way ANOVA on log transformed total MC-concentrations (to meet ANOVA requirements) in incubations with water from Lesser Prespa I indicated a significant treatment effect (*p* < 0.001) but no temperature effect (*p* = 0.074) and no treatment x temperature interaction (*p* = 0.240). Tukey’s post-hoc comparison revealed two homogeneous groups: 1) the controls and the +P treatments, 2) the +N and the +N+P treatments (Figure 4c). For log transformed total MC-concentrations in incubations with water from Lesser Prespa II the two-way ANOVA also indicated a significant treatment effect (*p* < 0.001) and no temperature (*p* = 0.076) or treatment × temperature interaction effect (*p* = 0.066). Tukey’s post-hoc comparison revealed three homogeneous groups: 1) the controls and the +P treatments, 2) the +N and 3) the +N+P treatments (Figure 4d).

**Figure 5:**
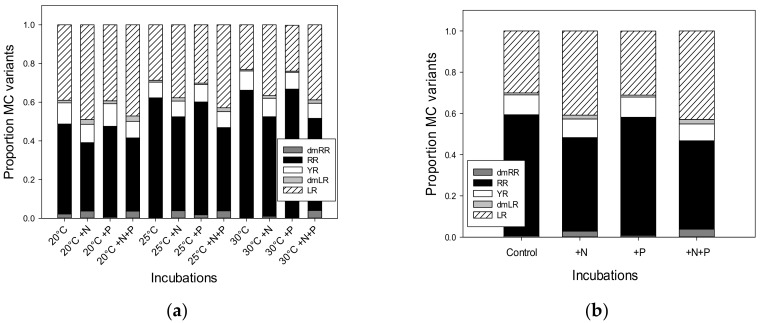
Pooled mean proportions of five different microcystin variants in the total MC pool in surface water taken from two locations in Vromolimni pond and two in lake Lesser Prespa after seven days incubation at regular (20 °C), high (25 °C) and extreme (30 °C) temperatures without additional nutrients addition, solely N-addition (+N), solely P-addition (+P) and both N+P addition (+N+P) (Panel **a**) and also pooled over temperatures (Panel **b**).

A closer inspection of the different MC variants showed that MC-RR had a tendency to become relatively more abundant at warmer temperatures, while MC-LR tended to be more abundant in the +N and +N+P incubations (Appendix B). Pooling these data for the four sites clearly showed this pattern (Figure 5a). Non-normal data distribution could not be solved by transformation and further pooling of the three incubation temperatures maintained the pattern of less MC-RR in +N and +N+P incubations and more MC-LR in the MC-pool (Figure 5b). This was confirmed by Kruskal-Wallis One Way Analysis of Variance on Ranks indicating significant differences among treatments in MC-RR proportions (*p* < 0.001) and in MC-LR proportions (*p* < 0.001). For both variants, a pairwise multiple comparison (Dunn's Method) revealed two homogeneous groups: 1) the non-treated (Control) and +P and 2) the +N and +N+P enriched treatments (Figure 5b). Hence, +N or +N+P treatments resulted in relatively less MC-RR and relatively more MC-LR.

## 3. Discussion

The results of our study are partly in agreement with the hypothesis that warming and nutrient additions promote cyanobacteria. Warming by itself did not promote cyanobacteria. In fact, in all incubations without any nutrient addition or in those with only P addition, cyanobacterial growth rates were negative meaning a decline in cyanobacterial chlorophyll-a concentrations over the incubation period. Concomitantly, eukaryotic algal growth rates were either positive (Vromolimni samples) or less negative (Lake Lesser Prespa samples). These results seem not to be in line with the proposed direct warming effect on cyanobacterial growth rates that would give cyanobacteria a competitive advantage over eukaryotic competitors at elevated temperatures [10,11]. In presence of additional nutrients (+N or +N+P), however, cyanobacterial growth rates were positive but still eukaryotic algal growth rates were higher, even at an incubation temperature of 30 °C (see Appendix A). Consequently, the percentage of cyanobacterial chlorophyll had been reduced in Vromolimni water from 85% at the start to on average of 71% (±8%) in +N and +N+P treatments and to 46% (±10%) in control and +P treatments. Likewise, in Lake Lesser Prespa water the percentage of cyanobacterial chlorophyll-a had been reduced from 60% at the start to on average of 50% (±13%) and 36% (±8%), respectively. A possible explanation might be that the flasks were continuously shaken therewith preventing sedimentation of non-cyanobacterial species and inhibiting buoyancy controlled positioning of the cyanobacteria. The vast majority of the cyanobacteria in the lake and pond water were *Microcystis* sp. with an undergrowth of *Dolichospermum* sp., both are known to express buoyancy control [19]. The difference between cyanobacterial and eukaryotic algal growth rates deviates from recent findings with water from a eutrophic urban pond, where cyanobacterial growth rates were higher than eukaryotic algal growth rates under non-treated and +N+P treated conditions at similar incubation temperatures as those employed here [17]. Grazing on edible eukaryotic algae by an assemblage of microzooplankton was considered as possible explanatory factor [17] but clearly the differences in cyanobacterial growth rates between studies, where cyanobacteria can be viewed as rather grazing resistant [20], indicate that the negative growth rates in non-treated and +P treated incubations in our experiment likely have another cause. 

The growth stimulation in +N and +N+P treatments evidently demonstrated N-limitation in all four water types used. Likewise, N-limitation was indicated by the molar DIN:P of 0.7–10 in the four water samples [18]. N-limitation in cyanobacteria will cause the breakdown of chlorophyll and phycocyanin, which is a major antenna pigment [21]. Such chlorosis [22] may result in the lower PHYTO-PAM detected cyanobacterial chlorophyll concentrations in non-treated and +P treated incubations. Interestingly, while the cyanobacterial chlorophyll concentrations had dropped to 24% (±9%) of the initial values, MC concentrations in non-treated and +P treated Vromolimni incubations were on average 37% (±32%) higher than at start. Similarly, in non-treated and +P treated Lake Prespa incubations MC concentrations were on average 20% (±47%) higher than at start, while the cyanobacterial chlorophyll concentrations had dropped to 29% (±9%) of the initial values. In general, MC quota might drop in response to N-limitation [23], however, the more MC producing strains could also be best protected against nutrient stress [24]. Under the N-limiting conditions, the diazotrophic *Dolichospermum* sp. could also be favoured, unlike non-diazotrophic cyanobacteria. Evidently, the incubation of non-treated and +P treated water did not lead to lower MC concentrations, yet whether this is caused by promotion of certain more toxic strains at the expense of non-toxic strains or species cannot be deciphered from our data. Therefore, additional experiments can be recommended that not only include the cyanobacterial community composition under the various scenarios but also could shed light on the shares of toxic and nontoxic cells [15]. 

Compared to initial concentrations, in +N and +N+P treated Vromolimni water on average 297% and 352% higher cyanobacterial chlorophyll concentrations were measured, while in Lake Lesser Prespa water these concentrations were 152% and 888% higher respectively. Hence, cyanobacterial concentrations were strongly enhanced by N additions and even further by combined N and P addition bringing them into a range where rapid scum formation could occur [25]. Such surface accumulations, particularly when blown to the lee side shore, can within several hours increase toxin concentrations by a factor of 1000 or more [25]. Here, adding N or both N and P to the Vromolimni and Lesser Prespa water resulted in significantly higher MC concentrations, which is straightforwardly explainable from higher cyanobacterial biomass in these incubations. However, no clear temperature effect on total MC concentrations was discernible. In none of the four water samples treated with nutrients did MC concentrations increase with higher temperatures. Greater temperatures could lead to a larger proportion of toxic cells in *Microcystis* field populations [15] but this does not automatically imply elevated MC concentrations, because surface water MC concentrations depend on the cyanobacterial biomass, the proportion of toxic cells and on MC cell quotas [16]. The MC cell quotas are influenced by environmental factors such as temperature, where elevated temperatures generally result in lower MC cell quota [17,26,27,28,29].

Adding N to the waters (either as +N, or +N+P) clearly changed the MC variant composition in favour of MC-LR at the expense of MC-RR. This response occurred in all four waters (Appendix B) and is not in support of the hypothesis that enhanced nitrogen availability would increase arginine rich MC variants like MC-RR [30]. Differences in cyanobacteria community composition are a likely explanation where adding N could promote non-diazotrophic *Microcystis*, while N-limitation could favour *Dolichospermum* sp. [31]. MC profiles are also dependent on environmental conditions, as for instance *Dolichospermum* sp. could produce relatively more MC-RR at higher temperatures [27]. Such pattern is found in the non-treated incubations in our experiment where the percentage of MC-RR in the total MC-pool at 20 °C was 47 (±5) % but increased to on average 62 (±3) % at 25 °C and 66 (±8) % at 30 °C (Appendix B). Since MC-LR is several times more toxic than the RR or desmethyl variants [32], further nitrogen enrichment seems to increase the ecological and public health risks associated with MCs via increased biomass of MC producers and a (slight) shift towards more toxic variants.

The observed N-limitation is fairly common during the growing season in eutrophic systems [33]. The fertilizers used on the agricultural area (bean fields) near the Prespa Lake system, such as YaraMila™ (12% N, 11% P_2_O_5_), TimacAgro Duofertil (11% N, 10% P_2_O_5_) or other Yara formulations (11% N, 15% P_2_O_5_), clearly contain more P relative to N (N:P ≈ 2.5) than the Redfield ratio (N:P of 7:1) [34]. The surface irrigation with water from Lesser Prespa will undoubtedly transport part of the nutrients into the lake. Consequently, in the Lesser Prespa system the N-limitation may be a result of relative over-enrichment with phosphorus from the common fertilizers used, whereas most of the added N will be taken up by bean plants and further be subjected to denitrification during transport in the ditches and in the reed beds adjacent to the lake [35]. Restricting the use of fertilizers is definitely advisable and evidently further N enrichment should be avoided. The enrichments experiments employed here, however, should not be used to indicate what element should be reduced to mitigate eutrophication as has been suggested in the literature [36]. The lake and pond have already been enriched, where such bioassays only reveal what might happen under further enrichments. 

Noteworthy are the increasing numbers of breeding colonies of pelicans, especially in Vromolimni Pond but also at the northern part of Lake Lesser Prespa [37], which, because of their predominantly out of the lake system foraging behaviour, will definitely have a considerable impact on further enrichment of the waters. Estimates of the nutrient (N and P) inputs of Dalmatian Pelicans, Great White Pelicans, Great Cormorants and Pygmy Cormorants yielded minimal 1189 kg N and 1614 kg P during the whole breeding season into Lesser Prespa and minimally 1253 kg N and 1015 kg P into the much smaller Vromolimni Pond [4]. In this study, we already see that P-limitation does not occur in the waters of this isolated pond whereas N limitation has the strongest effect on algal growth. The constant deposition of droppings by the large populations of these piscivorous water birds results in a high P-input in this small pond and the lack of P limitation. Increased P-input is expected also to occur at Lake Lesser Prespa near sites where water bird colonies are present. In our experiment +P-treatment of the lake water is found to have a strong effect on algae after +N-treatment indicating a co-limitation by N and P. So, for Lesser Prespa the strongest negative effect on water quality can be expected if both N and P levels increase.

Hence, in deciphering what should be controlled and how to mitigate eutrophication, a thorough diagnosis, or system analysis, is required [38]. In that view, also the observed decrease in wet-season precipitation and increase in number of dry years can be expected to further aggravate eutrophication symptoms [6]. Those climate change effects probably will exert a stronger impact on water quality deterioration than a direct warming effect on the cyanobacteria as shown from our warming incubations. Particularly, dry and warm summer periods will cause less water column mixing and can be expected to promote the development of surface accumulations of cyanobacteria [11]. When cyanobacteria are prevailing, these may locally lead to high risks associated with MCs [25] and, in fact, such accumulations have already been observed [5]. Hence, despite our incubations yielding no strong support for the proposition that warming and eutrophication may act in synergy [11,17], indirect warming effects most likely will further benefit cyanobacteria. 

The outcomes of our bioassays show that the Vromolimni Pond and Lake Lesser Prespa are vulnerable to further N enrichment. Given that the waters are already experiencing cyanobacterial blooms, measures not only to prevent further enrichment but also to reduce existing in-lake nutrient concentrations are needed. Large parts of Lake Lesser Prespa are probably characterized by a co-limitation of N and P. The presence of the water birds (via their droppings) have a vital role in provoking N-limitation at Vromolimni Pond and most likely in some parts of the lake too. Additionally, the role of internal loads of P and N from the lake sediments should also be taken into account.

## 4. Conclusions

Warming by itself did not enhance the algal production in the incubated seston from Lake Lesser Prespa or Vromolimni Pond. The findings show that warming of surface water from two sites in Lake Lesser Prespa to 25 °C or 30 °C led to elevated chlorophyll-a levels than the incubations at 20 °C when simultaneous addition by both nitrogen and phosphorus (+N+P) took place. However, no significant effect of warming on algal growth was found in single nutrient additions of either +N or +P. Warming of surface waters from two sites in Vromolimni Pond to 25 °C or 30 °C did not yield higher chlorophyll-a levels than incubations at 20°C if nutrients were added. Likewise adding P at 1.4 mg L^−1^ had no effect. The lake and pond water were N limited, because adding N at 14 mg L^−1^ or both N and P strongly promoted cyanobacteria and eukaryotic algae. MC concentrations in +N and both +N+P treated incubations were enhanced. No clear temperature effect on MC concentrations was found, however, in non-treated and +P treated incubations the share of the MC variant RR tended to increase with elevated temperature. In contrast, in +N and +N+P treated incubations RR was diminished and the more toxic variant MC-LR promoted. Lake Lesser Prespa and Vromolimni Pond are vulnerable to further +N treatment. The occurrence of cyanobacterial blooms indicates that not only external nutrient inputs should be reduced but also existing in-lake nutrient concentrations should be managed.

## 5. Materials and Methods 

### 5.1. Field Sampling 

**Figure 6:**
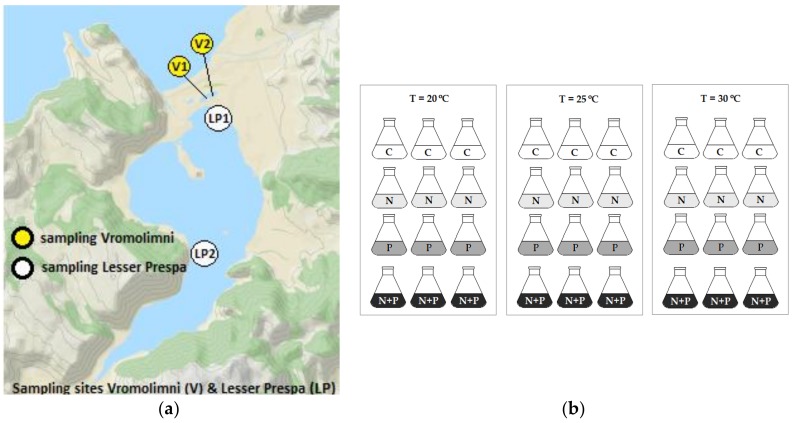
Sampling sites in Vromolimni Pond and Lake Lesser Prespa (Panel **a**) and the set-up overview of the algal growth assay in 2013 using surface water from the sampling locations LP1, LP2, V1 and V2 (Panel **b**); 36 flasks were incubated at three different temperatures (20, 25 and 30 °C) and received four different nutrient treatments - no nutrients addition/control (C), nitrogen addition (+N), phosphorus addition (+P) and addition of both nitrogen and phosphorus (+N+P), all in triplicate.

Surface water samples were collected on the 27th of September 2013 at two sites in the Greek part of Lake Lesser Prespa (40°41' N, 21°37' E) and two sites in Vromolimni Pond (Figure 6a). Per site, two litres of surface water (top 0.5 m) were collected in HDPE bottles. The samples were stored in a cool box and transported within 48 hours to the laboratory in Wageningen University (The Netherlands). In the laboratory, total- and cyanobacterial chlorophyll-a concentrations were measured using a PHYTO-PAM phytoplankton analyser (HeinzWalz GmbH, Effeltrich, Germany). Aliquots of each sample were filtered over a glass-fibre filter (Whatman GF/C, Whatman International Ltd., Maidstone, UK). The filters were stored at –20 °C for further MC analysis (see below) and the filtrates were analysed for dissolved inorganic nitrogen (DIN, i.e. ammonium and nitrate plus nitrite) and phosphate concentrations using a Skalar SAN++ Segmented Flow Analyser (Skalar Analytical B.V., Breda, The Netherlands) following the Dutch standard protocols [39,40,41]. A qualitative microscopic inspection for phytoplankton identification was performed using a Nikon light microscope (Nikon Instruments Europe BV, Amsterdam, The Netherlands).

### 5.2. Algal Growth Assay

The algal growth assay was set up five days after the sample’s collection at Lesser Prespa Lake and Vromolimni Pond. The aim was to assess the algal growth limiting nutrient(s) (Nitrogen, N; Phosphorus, P), to determine the growth of cyanobacteria and the production of microcystins (MCs) under regular (20 °C), high (25 °C) and extreme temperatures (30 °C) under non-enriched and nutrient-enriched conditions. To this end, 36 Erlenmeyer flasks were filled with 50 mL surface water from each sampling site. Nine of these flasks received no additional nutrients (no treatment/control – C; Figure 6b), to 9 flasks a 50 µL spike from a 1.4 g P L^−1^ K_2_HPO_4_ stock was added (1.4 mg P L^−1^; +P treatment), 9 other flasks received a 50 µL spike from a 14 g N L^−1^ NaNO_3_ stock (yielding 14 mg N L^−1^; +N treatment) and 9 other flasks received both a 50 µL P spike and a 50 µL N spike (+N+P treatment). All flasks were closed with cellulose plugs. Then, from each set of 9 similarly treated flasks per site, 3 flasks were incubated at a temperature of 20 °C, 3 flasks at 25 °C and the last 3 flasks at 30 °C, leading to 4 (sites) × 3 (temperatures) × 4 (nutrient treatments) × 3 (replicates) = 144 experimental units (Figure 6b). The selection of temperatures was based on an average water temperature of 20.1 °C recorded in Lake Lesser Prespa during April - September in 2013 [42], an average summer water temperature of 24.5 °C [42] and an extreme summer water temperature of 30 °C that has been recorded in the lake in July 2012 [43]. Each set of nutrient-treated flasks and controls were incubated for seven days in three Sanyo Gallenkamp (SANYO Electric Co., Ltd., Osaka, Japan) incubators set at 20 °C, 25 °C or 30 °C. Light was provided by fluorescent tubes that illuminated the flasks from above at 140 µmol photons m^−2^ s^−1^ in 18:6 h light:dark cycles. The flasks were shaken continuously at 60 rpm. Initially, and after 7 days, cyanobacterial and eukaryotic algae chlorophyll-a concentrations were measured using the PHYTO-PAM phytoplankton analyser (PHYTO-ED, system II version, Heinz Walz GmbH, Effeltrich, Germany). The chlorophyll-a concentration is considered a reliable measure of the response to eutrophication. Nonetheless, a second independent endpoint was included and the biovolume of samples after 7 days incubation was determined using an electronic particle counter (CASY cell counter (Schärfe System Gmbh., Reutlingen, Germany)). Aliquots of 10–30 mL from each flask were filtered over glass-microfiber filters (Whatman GF/C, Buckinghamshire, UK) and filters were stored individually in 8 mL glass tubes at –20 °C until MC analysis (see Section 5.3). 

### 5.3. Microcystin and Nodularin Analysis Using LC-MS/MS 

Frozen filters were extracted and processed for microcystin (MC) analysis as described in [44].Samples were analysed for eight MC variants (dm-7-MC-RR, MC-RR, MC-YR, dm-7-MC-LR, MC-LR, MC-LY, MC-LW and MC-LF) and nodularin (NOD) by Liquid Chromatography with tandem Mass Spectrometry detection (LC-MS/MS) as described in [44]. This LC-MS/MS analysis was performed on an Agilent 1200 LC and an Agilent 6410A QQQ (Waldbronn, Germany). The MS/MS settings of the eight different MC variants and NOD are given in Table 3 in [44]. Each MC variant was quantified against a calibration curve and subsequently corrected for recovery. The calibration curves were made using certified calibration standards obtained from DHI LAB Products (Hørsholm, Denmark) [44]. Information about the limits of detection (LOD) and quantification (LOQ) in samples analysed for microcystins (MC) and nodularin (NOD) by LC-MS/MS is presented in Table A1 (Appendix C), while other method performance characteristics are provided in [44].

### 5.4. Data Analysis 

Total- and cyanobacterial chlorophyll-a concentrations in the initial samples were analysed by one-way ANOVA followed by Holm-Sidak post-hoc comparison. Total- and cyanobacterial chlorophyll concentrations of the experiment would preferably have been analysed using three-way ANOVA (site, temperature, nutrients) or by two-way ANOVA per site (temperature and nutrient treatment as factors) but Shapiro-Wilk test for normality indicated non-normal distribution of data that could not be overcome by transformations (i.e., *p* < 0.05). Hence, a non-parametric Kruskal-Wallis One Way Analysis of Variance on Ranks was performed per site on treatments followed by a Tukey post-hoc test to detect differences between groups. MC concentrations or log transformed total MC-concentrations (to meet ANOVA requirements) were analysed running a two-way ANOVA for incubations from each site. Differences between groups were detected by Tukey’s post-hoc comparisons. Non-normal data distributions did not allow testing of the proportions of MC-variants by three- or two-way ANOVA. To this end, the percentages of MC-RR and MC-LR were analysed by Kruskal-Wallis One Way Analysis of Variance on Ranks on data pooled over sites and temperatures (thus only for nutrient treatments), followed by pairwise multiple comparisons (Dunn's Method). All analyses were done in the program SigmaPlot (version 13.0, Systat Software Inc., San Jose, CA, USA).

Chlorophyll-a based growth rates (d^−1^) were calculated from cyanobacterial- and eukaryote algal chlorophyll-a concentrations (μg L^−1^)assuming exponential growth of cyanobacteria and eukaryotic phytoplankton over the seven-day incubation period according to the mathematical equation provided by Lürling et al. 2017 [17] . 

## Figures and Tables

**Figure A1:**
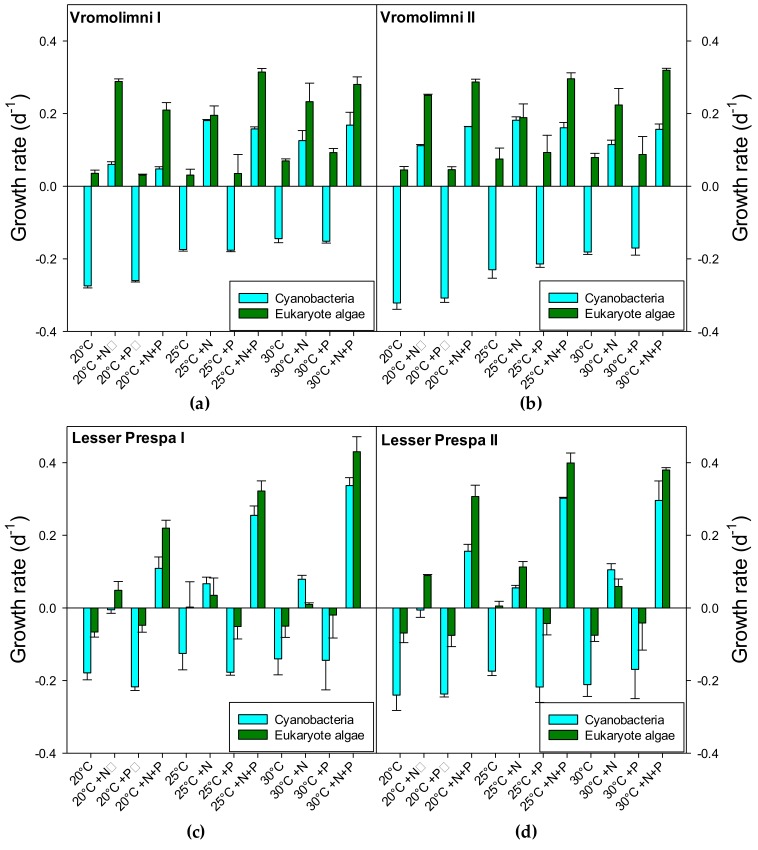
Cyanobacterial- (blue) and eukaryote algal (green) growth rates (d^−1^) based on PHYTO-PAM derived chlorophyll-a concentrations (µg L^−1^) in surface water taken from two locations in Vromolimni Pond (Panels **a** and **b**) and two in Lake Lesser Prespa (Panels **c** and **d**) that had been incubated for seven days at regular (20 °C), high (25 °C) and extreme (30 °C) temperatures without extra nutrients added (Control, C), solely N addition (+N), solely P-addition (+P) and both N+P addition (+N+P). Error bars indicate one standard deviation (*n* = 3).

**Figure A2:**
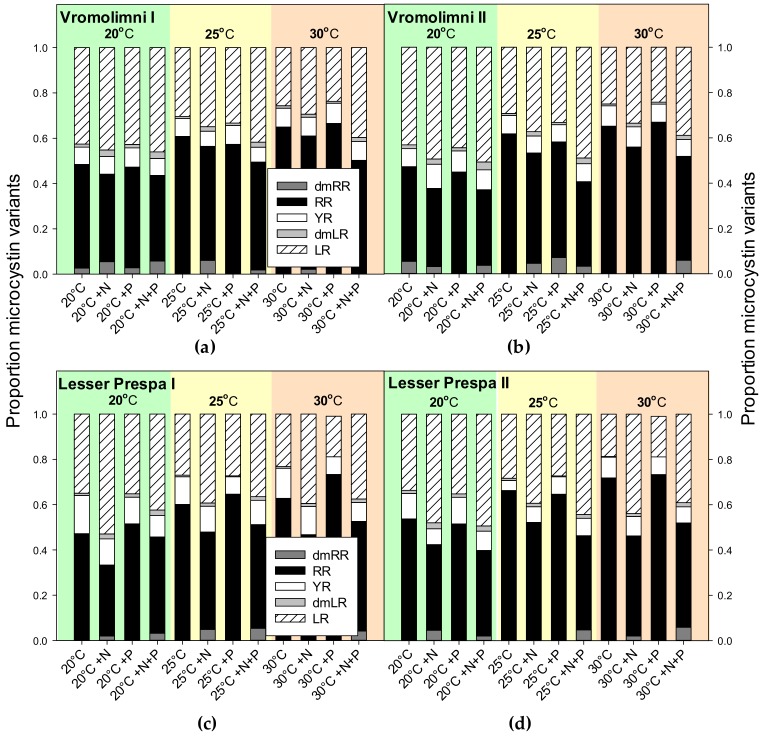
Mean proportions of five different microcystin (MC) variants in the total MC pool in surface water taken from two locations in Vromolimni Pond (Panels **a** and **b**) and two in Lake Lesser Prespa (Panels **c** and **d**) after seven days incubation at regular (20 °C), high (25 °C) and extreme (30 °C) temperatures without extra nutrients added, solely N-addition (+N), solely P-addition (+P) and both N+P addition (+N+P).

## Appendix A

The chlorophyll-a based growth rates for cyanobacteria and eukaryotic phytoplankton in the algal growth experiment (Figure A1).

## Appendix B

The proportions of the detected MC variants in the overall MC pool per treatment (Figure A2). The variants MC-RR and MC-LR were most abundant and varied per treatment, where in general MC-RR declined in +N and +N+P treatments, while MC-LR increased. The variant MC-YR was more conservatively present. The desmethyl variants dmMC-RR and dmMC-LR were more abundant in the +N and +N+P treated treatments (Figure A2). No hydrophobic MCs (MC-LY, MC-LW, MC-LF) nor NOD were detected.

## Appendix C

**Table A1:** Limits of detection (LOD) and quantification (LOQ) concentrations in the samples analysed for microcystins (MC) and nodularin (NOD) by LC-MS/MS.

Compound	LOD^1^ (µg L^−1^)	LOQ^2^ (µg L^−1^)
dm-7-MC-RR	0.253	0.253
MC-RR	0.187	0.187
NOD	<0.017	0.017
MC-YR	<0.024	0.024
dm-7-MC-LR	<0.023	0.023
MC-LR	<0.045	0.045
MC-LY	0.395	0.395
MC-LW	0.269	0.538
MC-LF	0.113	0.113

^1^ Limit of detection, ^2^ Limit of quantification.
